# Supplementary material for: The Invasion of Galinsoga quadriradiata into High Elevations Is Shaped by Variation in AMF Communities
Source: Plants (Basel). 2023 Sep 6;12(18):3190. doi: 10.3390/plants12183190 (PMC10534310; doi:10.3390/plants12183190)
Supplement: Supplementary file 1 [file plants-12-03190-s001.zip › plants-2534276-supplementary.pdf]

Supporting Information

The Invasion of *Galinsoga quadriradiata* into High Elevations Is Shaped by Variation in AMF Communities

Gang Liu [1,2,3,*,†], Ruiling Liu [1,†], Benjamin R. Lee [4,5,6], Xingjiang Song [1], Wengang Zhang [1], Zhihong Zhu [1,2,3] and Yan Shi [1]

[1] College of Life Sciences, Shaanxi Normal University, Xi'an 710119, China
[2] Research Center for UAV Remote Sensing, Shaanxi Normal University, Xi'an 710119, China
[3] Changqing Teaching & Research Base of Ecology, Shaanxi Normal University, Xi'an 710119, China
[4] Carnegie Museum of Natural History, Pittsburgh, PA 15213, USA
[5] Department of Biological Sciences, University of Pittsburgh, Pittsburgh, PA 15213, USA
[6] Holden Forest and Gardens, Kirtland, OH 44094, USA
* Correspondence: 2003liugang@163.com; Tel.: +86-131-8601-1103
† These authors contributed equally to this work.

Supplemental Tables

Table S1 Results of generalized linear mixed models (GLMMs) on the growth of *G. quadriradiata*. R:S, Root-Shoot ratio; LNC, leaf nitrogen concentration; LPC, leaf phosphorus concentration. Pop: population of *G. quadriradiata* (1-5). AMF: inoculated (AMF+) and uninoculated (AMF −). Inoculation source (InS): (S), inoculated with AMF spores from the same elevational population of *G. quadriradiata*; (NS), inoculated with AMF spores from a different elevational population of *G. quadriradiata*; (H), inoculated with AMF spores from the high-altitude site that has not yet been invaded by *G. quadriradiata*. Cul: mono- or polyculture. Competitors: the three native plants, *Achnatherum splendens*, *Medicago sativa*, and *Picris hieracioides*. CompTy: nitrogen-fixing or non-nitrogen-fixing native competitor. Effects were considered significant at p < 0.05, indicated by bold font.

**Effect**		**Pop**	**Cul**	**Pop×Cul**	**CompTy(Cul)**	**Pop×CompTy(Cul)**	**AMF**	**Pop×AMF**	**Cul×AMF**	**Pop×Cul×AMF**	**CompTy×AMF(Cul)**	**Pop×CompTy×AMF(Cul)**	**InS(AMF)**	**Pop×InS(AMF)**	**Cul×InS(AMF)**	**Pop×Cul×InS(AMF)**	**CompTy×InS(Cul×AMF)**	**Pop×CompTy×InS(Cul×AMF)**
Total mass	*df*	4,931	1,1	4,931	1,931	4,931	1,931	4,931	1,931	4,931	1,931	4,931	2,931	8,931	2,931	8,931	2,931	8,931
	F	35.62	101.80	10.69	0.01	2.93	49.85	14.44	124.42	4.70	4.86	1.59	15.99	7.14	4.61	3.45	3.81	2.64
	p	**<.0001**	0.063	**<.0001**	0.948	**0.020**	**<.0001**	**<.0001**	**<.0001**	**0.001**	**0.028**	0.176	**<.0001**	**<.0001**	**0.010**	**0.001**	**0.023**	**0.007**
R:S	*df*	4,931	1,1	4,931	1,931	4,931	1,931	4,931	1,931	4,931	1,931	4,931	2,931	8,931	2,931	8,931	2,931	8,931
	F	4.34	3.82	1.86	3.59	7.18	15.39	3.92	5.96	2.94	5.36	9.21	1.96	0.32	0.32	0.26	0.03	0.11
	p	**0.002**	0.301	0.116	0.058	**<.0001**	**<.0001**	**0.004**	**0.015**	**0.020**	**0.021**	**<.0001**	0.141	0.957	0.727	0.979	0.971	0.999
Seed mass	*df*	4,934	1,1	4,934	1,934	4,934	1,934	4,934	1,934	4,934	1,934	4,934	2,934	8,934	2,934	8,934	2,934	8,934

	F	61.46	249.71	12.22	0.03	2.66	157.87	29.50	172.31	7.77	5.28	1.40	11.05	8.81	4.34	3.25	1.69	2.45
	p	**<.0001**	**0.040**	**<.0001**	0.853	**0.032**	**<.0001**	**<.0001**	**<.0001**	**<.0001**	**0.022**	0.233	**<.0001**	**<.0001**	**0.013**	**0.001**	0.184	**0.013**
Seed mass ratio	*df*	4,931	1,1	4,931	1,931	4,931	1,931	4,931	1,931	4,931	1,931	4,931	2,931	8,931	2,931	8,931	2,931	8,931
	F	22.12	65.14	1.06	2.90	0.30	102.88	3.67	10.72	1.29	1.65	0.87	1.35	12.18	4.25	2.59	0.08	1.89
	p	**<.0001**	0.079	0.376	0.089	0.876	**<.0001**	**0.006**	**0.001**	0.270	0.199	0.479	0.260	**<.0001**	**0.015**	**0.009**	0.9261	0.059
Number of capitula	*df*	4,934	1,1	4,934	1,934	4,934	1,934	4,934	1,934	4,934	1,934	4,934	2,934	8,934	2,934	8,934	2,934	8,934
	F	46.80	159.44	9.28	0.26	2.19	66.07	19.84	100.23	6.33	4.20	0.91	33.32	11.78	6.37	3.94	1.80	2.07
	p	**<.0001**	0.050	**<.0001**	0.613	0.069	**<.0001**	**<.0001**	**<.0001**	**<.0001**	**0.041**	0.455	**<.0001**	**<.0001**	**0.002**	**<.0001**	0.166	**0.036**
LNC	*df*	4,636	1,1	4,636	1,636	4,636	1,636	4,636	1,636	4,636	1,636	4,636	2,636	8,636	2,636	8,636	2,636	8,636
	F	17.86	23.47	3.33	0.90	1.14	13.26	8.83	60.18	5.05	0.07	3.44	107.05	8.80	4.13	3.65	0.36	1.24
	p	**<.0001**	0.130	**0.010**	0.345	0.335	**0.0003**	**<.0001**	**<.0001**	**0.001**	0.786	**0.009**	**<.0001**	**<.0001**	**0.017**	**0.0004**	0.696	0.271
LPC	*df*	4,636	1,1	4,636	1,636	4,636	1,636	4,636	1,636	4,636	1,636	4,636	2,636	8,636	2,636	8,636	2,636	8,636
	F	1.16	1.62	1.28	0.07	1.91	125.61	1.43	0.01	2.43	0.49	1.00	5.37	3.16	2.43	4.50	0.59	1.57
	p	0.327	0.423	0.278	0.792	0.107	**<.0001**	0.221	0.943	**0.047**	0.485	0.409	**0.005**	**0.002**	0.089	**<.0001**	0.556	0.131
AMF colonization rate	*df*	4,637	1,1	4,637	1,637	4,637	1,637	4,637	1,637	4,637	1,637	4,637	2,637	8,637	2,637	8,637	2,637	8,637
	F	3.53	1.22	0.78	0.03	0.24	26.77	0.89	5.38	0.12	0.12	0.17	19.07	2.36	1.20	1.06	0.58	0.20
	p	**0.007**	0.469	0.541	0.854	0.913	**<.0001**	0.471	**0.021**	0.977	0.734	0.956	**<.0001**	**0.017**	0.303	0.389	0.560	0.991

Table S2 Results of generalized linear mixed models (GLMMs) on the MD (mycorrhizal dependency) of *G. quadriradiata*. Pop: population of *G. quadriradiata* (1-5). AMF: inoculated (AMF+) and uninoculated (AMF−). Inoculation source (InS): (S), inoculated with AMF spores from the same elevational population of *G. quadriradiata*; (NS), inoculated with AMF spores from a different elevational population of *G. quadriradiata*; (H), inoculated with AMF spores from the high-altitude site that has not yet been invaded by *G. quadriradiata*. Cul: mono- or polyculture. Competitors: the three native plants, *Achnatherum splendens*, *Medicago sativa*, and *Picris hieracioides*. CompTy: nitrogen-fixing or non-nitrogen-fixing native competitor. Fixed factors: Pop, Cul, CompTy nested in culture and InS; random factor: Competitors nested in Cul. Effects were considered significant at $p < 0.05$, indicated by bold font.

**Effect**		**Pop**	**Cul**	**Pop×Cul**	**CompTy(Cul)**	**Pop×CompTy(Cul)**	**InS**	**Pop×InS**	**Cul×InS**	**Pop×Cul×InS**	**CompTy×InS(Cul)**	**Pop×CompTy×InS(Cul)**
	df	4,747	1,1	4,747	1,747	4,747	2,747	8,747	2,747	8,747	2,747	8,747
MD	*F*	25.26	132.40	14.28	4.60	3.13	6.04	2.66	1.27	0.78	4.56	3.46
	p	**<.0001**	0.055	**<.0001**	**0.032**	**0.014**	**0.003**	**0.007**	0.281	0.623	**0.011**	**0.001**

Table S3 Results of generalized linear mixed models (GLMMs) for total mass and RII (relative interaction index) of invasive vs. native plant species. AMF: inoculated (AMF+) and uninoculated (AMF−). Species types (SpTy): invasive or native. Inoculation source for native species (InSna): either inoculated with AMF collected from a site within the invaded range or inoculated with AMF spores from the high-elevation site that has not yet been invaded by *G. quadriradiata*. Cul: mono- or polyculture. Competitors: random effect of native species (*Achnatherum splendens*, *Medicago sativa*, and *Picris hieracioides*). CompTy: random effect of nitrogen-fixing or non-nitrogen-fixing native species. For the total mass, fixed factors: SpTy, Cul, and InSna nested in AMF; random factors: Competitors nested in culture and CompTy nested in culture. For the RII, fixed factors: SpTy, and InSna nested in AMF; random factor: Competitors nested in culture. Effects were considered significant at $p < 0.05$, indicated by bold font.

**Effect**		**SpTy**	**Cul**	**SpTy×Cul**	**InSna(AMF)**	**SpTy×InSna(AMF)**	**Cul×InSna(AMF)**	**SpTy×Cul×InSna(AMF)**
Total mass	*df*	1,1919	1,1919	1,1919	2,1919	2,1919	2,1919	2,1919
	F	222.32	141.64	5.58	134.56	14.74	187.45	4.29
	p	**< .0001**	**< .0001**	**0.018**	**< .0001**	**< .0001**	**< .0001**	**0.014**
RII	*df*	1,1466			2,1466	2,1466		

F	45.68	261.77	2.93
p	**< .0001**	**< .0001**	0.054

Table S4 Results of generalized linear mixed models (GLMMs) on the total mass and RII (relative interaction index) for the three native plant species. Abbreviations are as described in Table S3.

**Effect**		**Sp**	**Cul**	**Sp×Cul**	**InSna(AMF)**	**Sp×InSna(AMF)**	**Cul×InSna(AMF)**	**Sp×Cul×InSna(AMF)**
	df	2,923	1,4	2, 923	2,923	4,923	2,923	4,923
Total mass	*F*	190.22	185.09	98.60	304.42	56.27	352.32	82.74
	p	**< .0001**	**0.0002**	**< .0001**	**< .0001**	**< .0001**	**< .0001**	**< .0001**
	df	2,722			2,722	4,722		
RII	*F*	3.82			188.95	13.15		
	p	**0.023**			**< .0001**	**< .0001**		

Table S5 Results of generalized linear mixed models (GLMMs) for LNC (leaf nitrogen concentration) and LPC (leaf phosphorus concentration) of *G. quadriradiata*. Driver abbreviations are as described in Table S3

**Effect**		**CompTy**	**AMF**	**CompTy×AMF**	**InS(AMF)**	**CompTy×InS(AMF)**
	df	1,514	1,514	1,514	2,514	2,514
LNC	*F*	0.82	7.19	0.07	44.68	0.27
	P	0.366	**0.008**	0.795	**< .0001**	0.762
	df	1,514	1,514	1,514	2,514	2,514
LPC	*F*	0.07	88.93	0.39	9.06	0.51
	P	0.784	**< .0001**	0.531	**< .0001**	0.600

Table S6 Location and elevation of *Galinsoga quadriradiata* populations and AMF inoculation sources.

Site ID	Latitude (N°)	Longitude (E°)	Elevation (m)	Soil	Pop	AMF sources
1	33.2973	108.1823	573	Soil 1	Pop 1	AMF+1
2	32.0915	109.2566	1064	Soil 2	Pop 2	AMF+2
3	33.4342	108.4457	1526	Soil 3	Pop 3	AMF+3
4	32.0278	109.3192	1739	Soil 4	Pop 4	AMF+4
5	32.0238	109.3320	1930	Soil 5	Pop 5	AMF+5
6	33.4827	108.4863	2391	Soil H		AMF+H

Table S7 Physical and chemical properties of planting soil. TN: Soil total nitrogen concentration; TP: Soil total phosphorus concentration; NH_4^+-N: Soil ammonium-nitrogen concentration; NO_3^--N: Soil nitrate-nitrogen concentration; AP: Soil available phosphorus concentration. Values represent means ± standard error.

**TN** mg / g	**TP** mg / g	**NH_4^+-N** mg / kg	**NO_3^--N** mg / kg	**AP** mg / kg
3.452 ± 0.156	0.726 ± 0.020	10.555 ± 0.886	22.825 ± 1.121	22.021 ± 0.848

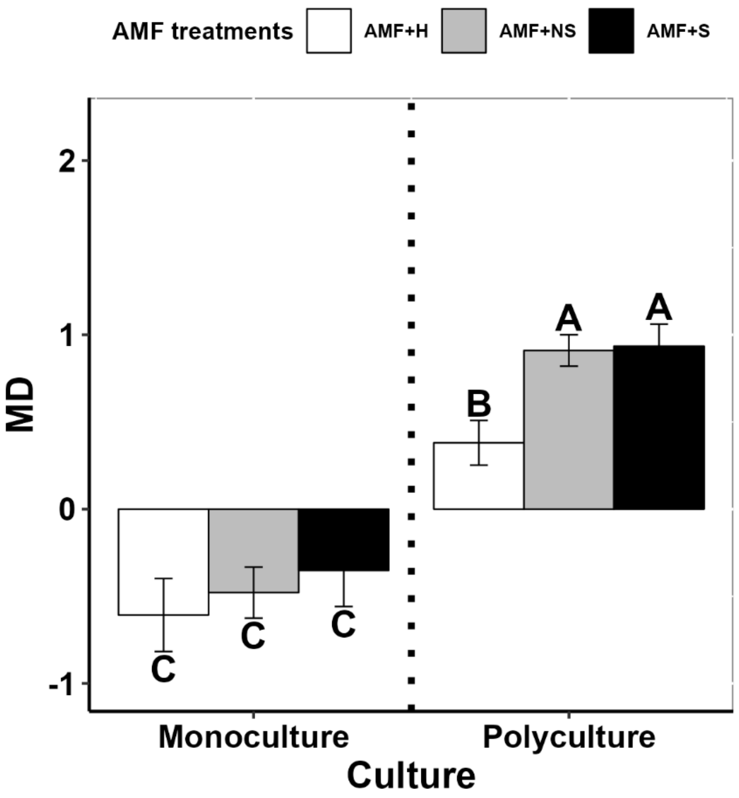

Figure S1 The effects of different treatments on MD (mycorrhizal dependency) of *Galinsoga quadriradiata* grown in either mono- or polyculture. AMF treatments: AMF+S, inoculated with AMF collected from the same site of the invasive plant population; AMF+NS, inoculated with AMF collected from invaded sites from different elevations (all four NS sites combined); AMF+H, inoculated with AMF collected from the high-elevation, uninvaded site. Letters indicate significant difference ($p < 0.05$) and error bars show ± standard error around mean values.

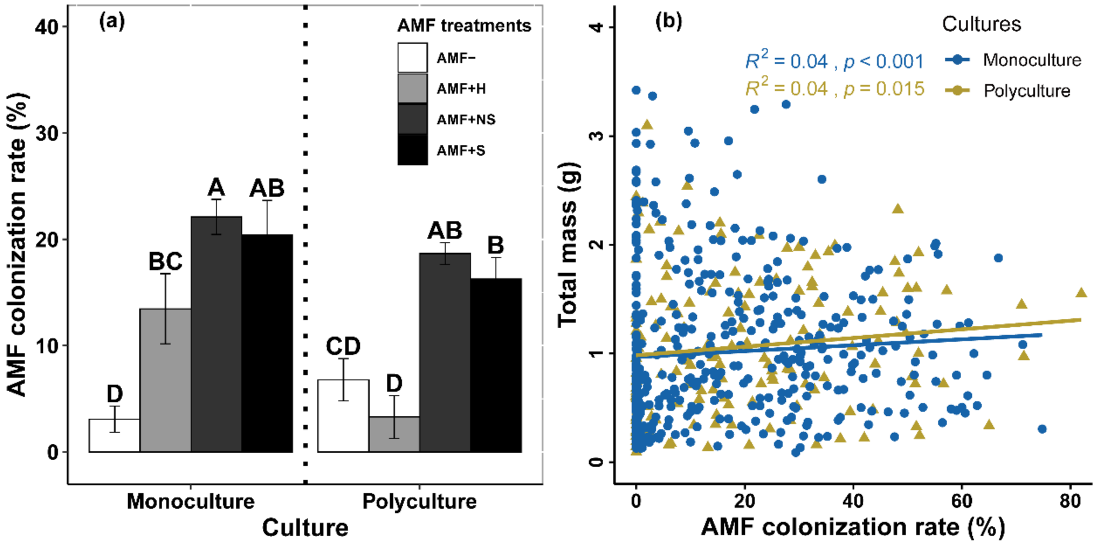

Figure S2 (a) The effects of different AMF inoculation treatments on AMF colonization rate of *Galinsoga quadriradiata* grown in either mono- or polyculture. Culture: Monoculture, one plant in a pot; Polyculture, a native and an invasive plant cultivated in a pot. AMF treatments: AMF－, uninoculated; AMF+S, inoculated with AMF collected from the same site of the invasive plant population; AMF+NS, inoculated with AMF collected from invaded sites from different elevations (all four NS sites combined); AMF+H, inoculated with AMF collected from the high-elevation, uninvaded site. Letters indicate significant difference (p < 0.05) and error bars show ± standard error around mean values. (b) Correlation between total biomass and AMF colonization rate for *G. quadriradiata* grown in mono- vs. polyculture. R^2 and p values are indicated in the figure.

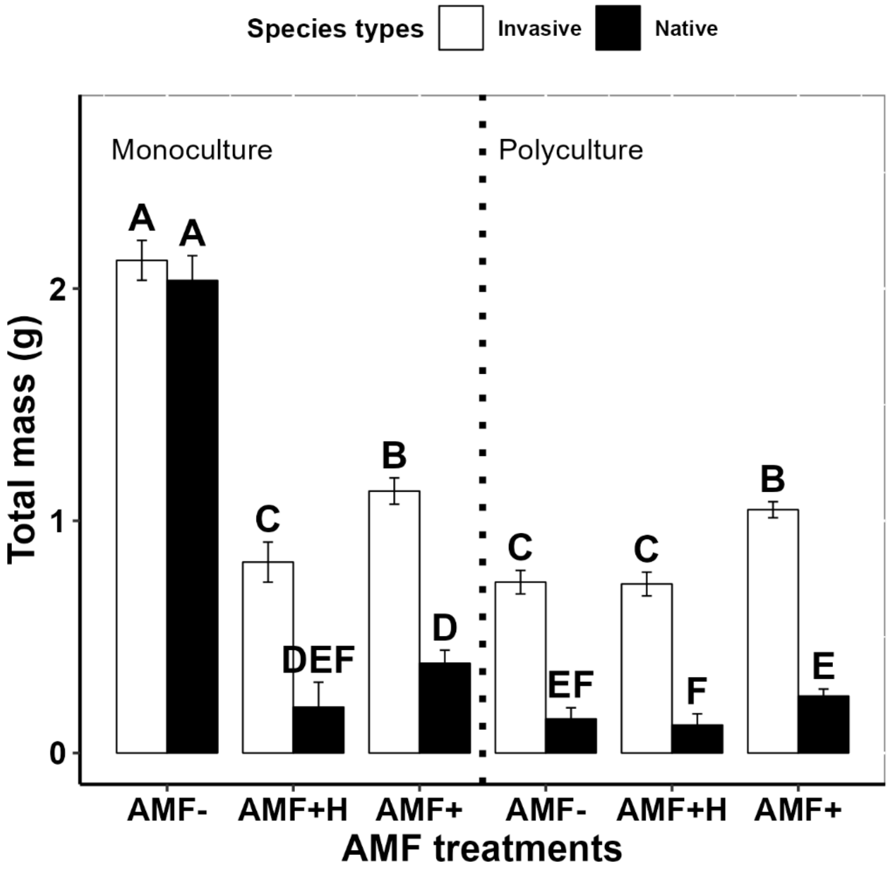

Figure S3 Effects of AMF inoculation treatment on total mass of invasive plant (white) versus native (black) plant species growing in either mono- or polyculture. AMF treatments, letters, and error bars are as described in Figure S1.

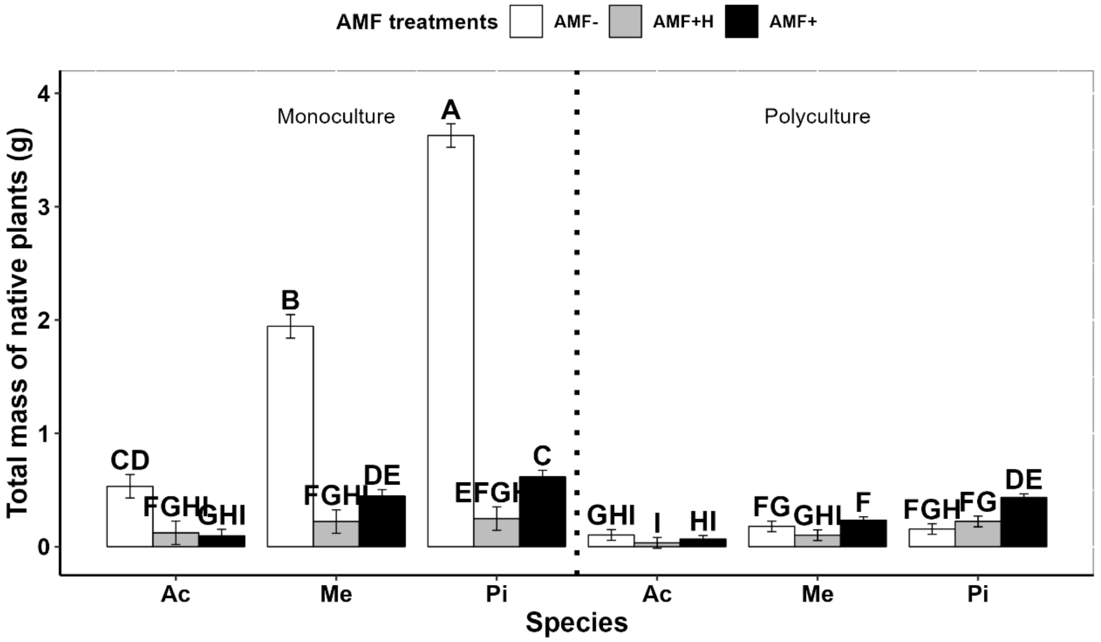

Figure S4 Effects of AMF inoculation treatment on the total mass of the three native plant species growing in either mono- or polyculture: *Achnatherum splendens* (Ac), *Medicago sativa* (Me), and *Picris hieracioides* (Pi). AMF treatments, letters, and error bars are as described in Figure S1.

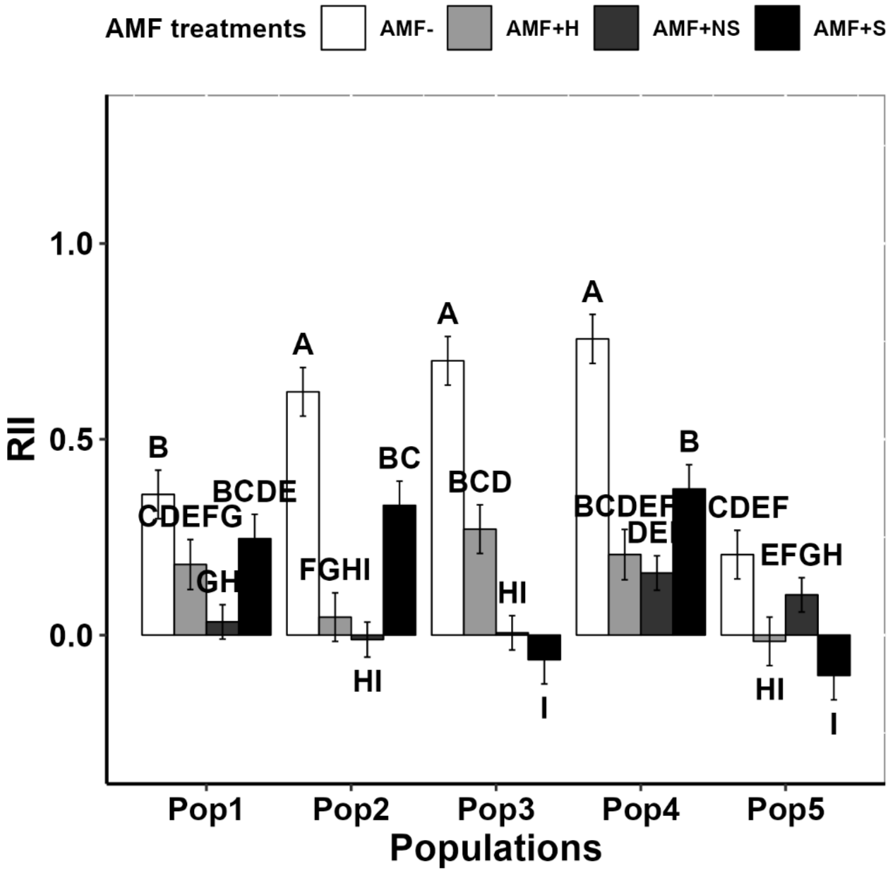

Figure S5 Effects of different AMF treatments on the RII (relative interaction index) of *Galinsoga quadriradiata* sourced from populations at different elevations. AMF treatments: AMF−, uninoculated; AMF+S, inoculated with AMF collected from the same site of the invasive plant population; AMF+NS, inoculated with AMF collected from invaded sites from different elevations (all four NS sites combined); AMF+H, inoculated with AMF collected from the high-elevation, uninvaded site. Populations range from low (Pop1) to high (Pop5) elevations, as described in Table S6. Letters indicate significant difference ($p < 0.05$) and error bars show ± standard error around mean values.

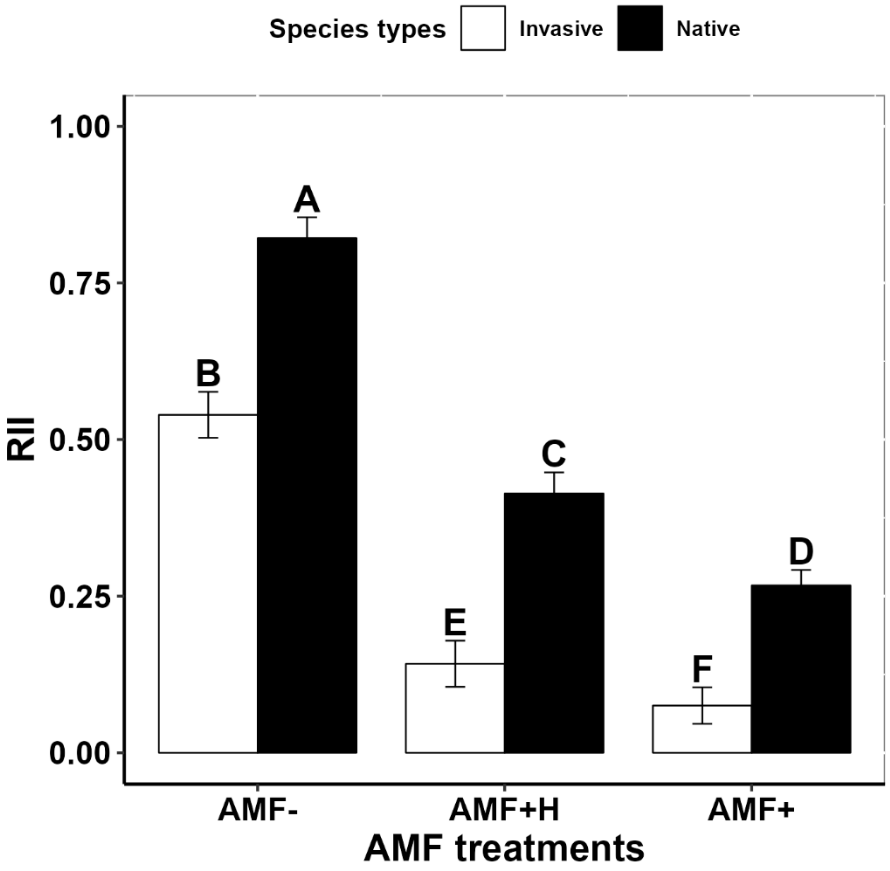

Figure S6 Effects of AMF inoculation treatments on the RII (relative interaction index) of invasive (white bars) and native (black bars) plants grown in polyculture. AMF treatments: AMF−, uninoculated; AMF+, includes AMF+S and AMF+NS treatments (as described for Figure S1); AMF+H, inoculated with AMF collected from the high-elevation, uninvaded site. Letters and error bars are as described in Figure S1.

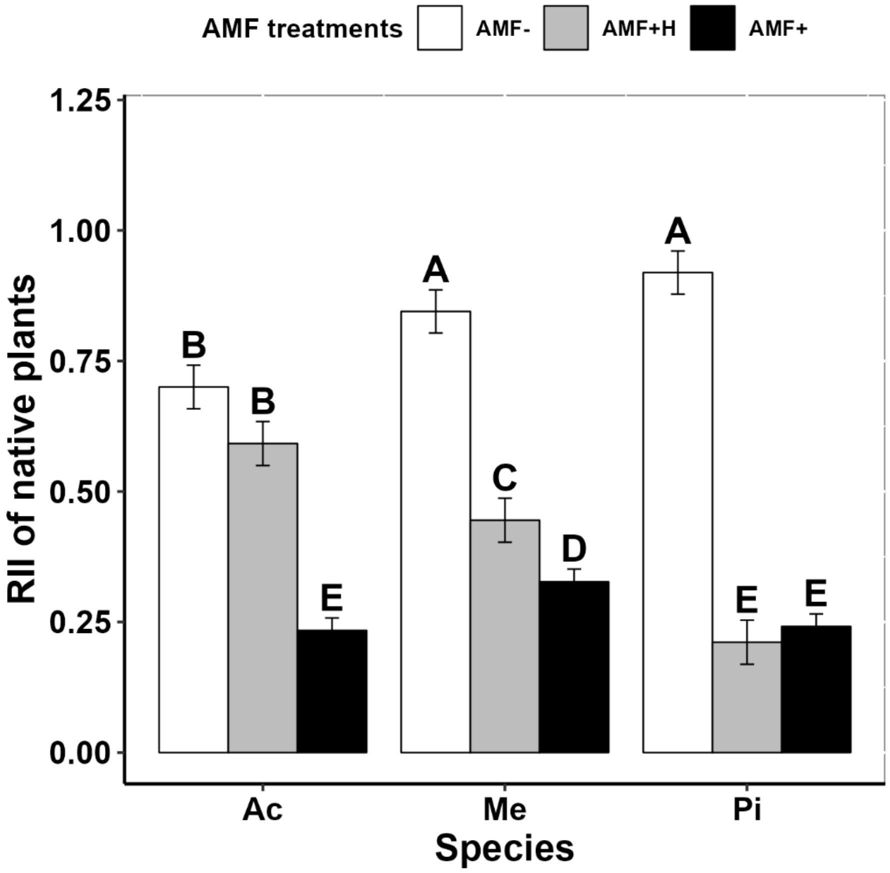

Figure S7 Effects of AMF inoculation treatments on the RII (relative interaction index) of three native plants grown in polyculture with invasive *G. quadriradiata*. AMF treatments, lettering, and error bars are as described in Figure S1. Species abbreviations are as described in Figure S4.

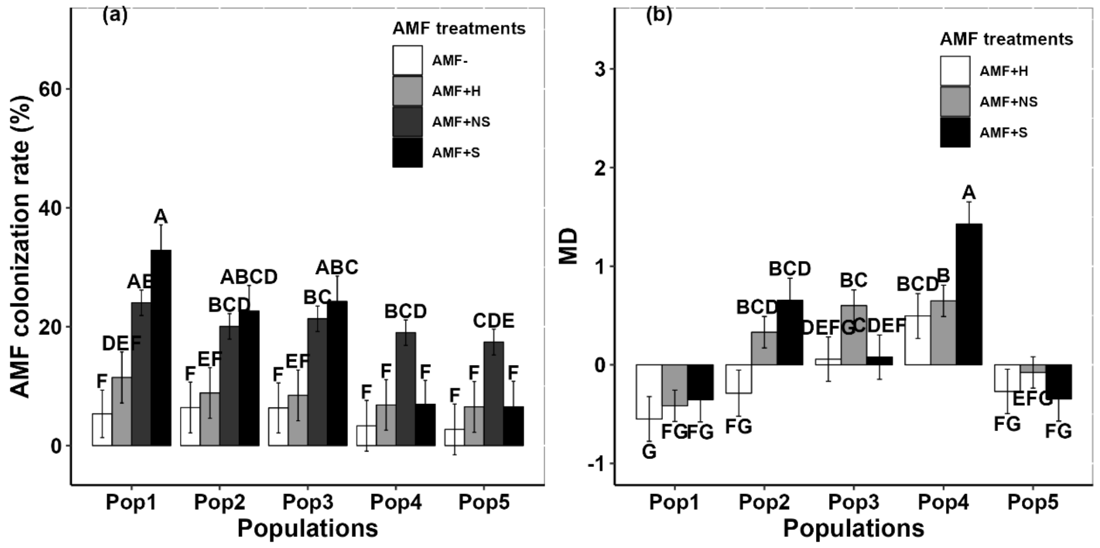

Figure S8 Effects of different AMF inoculation treatments on (a) AMF colonization rate and (b) mycorrhizal dependency (MD) of *Galinsoga quadriradiata* sourced from populations ranging from low elevations (Pop1) to high (Pop5; see Table S6 for elevation information). AMF inoculation treatments, lettering, and error bars are as described in Figure S1.

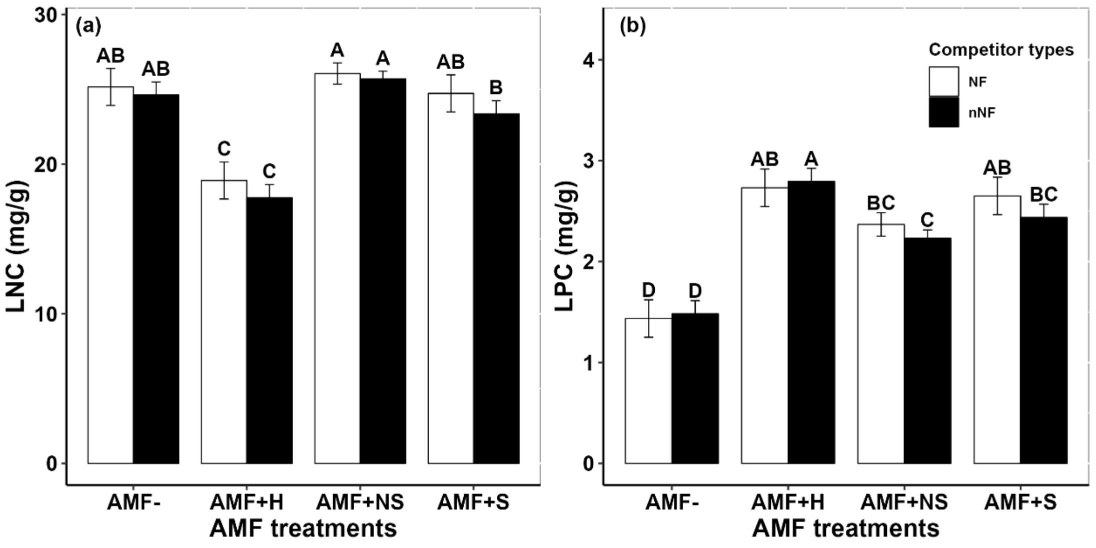

Figure S9 Effect of competitor types (nitrogen-fixing (white bars) or non-nitrogen-fixing (black bars)) on *Galinsoga quadriradiata* (a) leaf nitrogen concentration (LNC) and (b) leaf phosphorus concentration (LPC) when grown in polyculture with native competitors. AMF treatments, lettering, and error bars are as described in Figure S1.

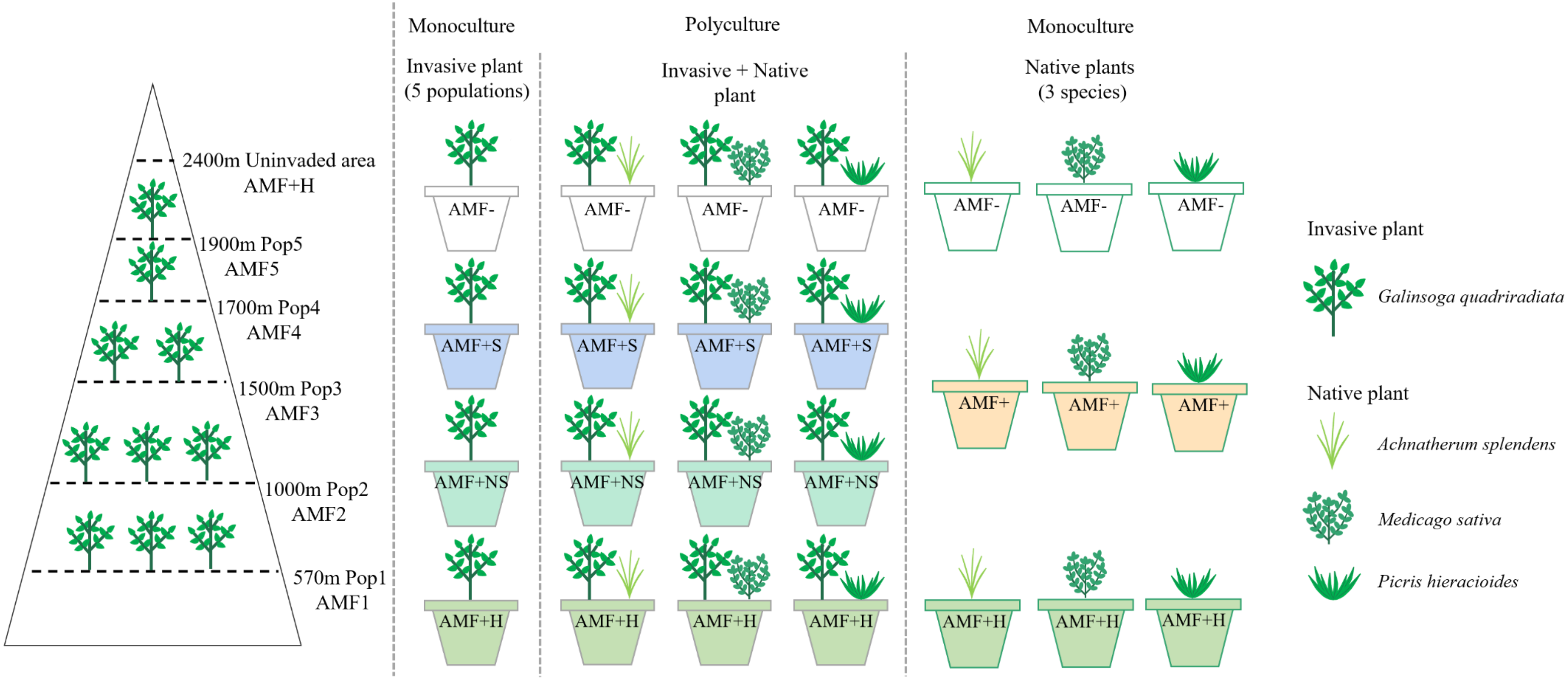

Figure S10 The distribution of the invasive plant populations and AMF source, and the experimental design for the greenhouse experiment. In both monoculture and polyculture, all five populations of *Galinsoga quadriradiata* were cultured following the design. AMF treatments: AMF−,

uninoculated; AMF+S, inoculated with AMF collected from the same site of the invasive plant population; AMF+NS, inoculated with AMF collected from invaded sites from different elevations (all four NS sites combined); AMF+H, inoculated with AMF collected from the high-elevation, uninvaded site. We had 10 replicates for three AMF treatments [AMF−, AMF+S and AMF+H: 5 invasive populations × 3 inoculation treatments × (1 monoculture pot + 3 mixed species pots) × 10 replicates = 600 pots], and 5 replicates for other four AMF treatments [AMF+NS: 5 invasive populations × 4 inoculation treatments × (1 monoculture pot + 3 mixed species pots) × 5 replicates = 400 pots], total 1000 pots for the invasive plant. We had 3 native plants × 7 inoculation treatments × 10 replicates = 210 pots for the native plants grown in monoculture. In total, we had 1210 pots and 1960 individual plants.
